# Metabolomics and random forests in patients with complex congenital heart disease

**DOI:** 10.3389/fcvm.2022.994068

**Published:** 2022-10-05

**Authors:** Miriam Michel, Kai Thorsten Laser, Karl-Otto Dubowy, Sabine Scholl-Bürgi, Erik Michel

**Affiliations:** ^1^Division of Pediatrics III – Cardiology, Pulmonology, Allergology and Cystic Fibrosis, Department of Child and Adolescent Health, Medical University of Innsbruck, Innsbruck, Austria; ^2^Division Pediatrics I – Inherited Metabolic Disorders, Department of Child and Adolescent Health, Medical University of Innsbruck, Innsbruck, Austria; ^3^Center of Pediatric Cardiology and Congenital Heart Disease, Heart and Diabetes Center North Rhine-Westphalia, Ruhr-University of Bochum, Bad Oeynhausen, Germany; ^4^Clinic for Pediatrics, Medizin Campus Bodensee, Friedrichshafen, Germany

**Keywords:** congenital heart disease, Fontan, metabolomics, random forest, statistics

## Abstract

**Introduction:**

It is increasingly common to simultaneously determine a large number of metabolites in order to assess the metabolic state of, or clarify biochemical pathways in, an organism (“metabolomics”). This approach is increasingly used in the investigation of the development of heart failure. Recently, the first reports with respect to a metabolomic approach for the assessment of patients with complex congenital heart disease have been published. Classical statistical analysis of such data is challenging.

**Objective:**

This study aims to present an alternative to classical statistics with respect to identifying relevant metabolites in a classification task and numerically estimating their relative impact.

**Methods:**

Data from two metabolomic studies on 20 patients with complex congenital heart disease and Fontan circulation and 20 controls were reanalysed using random forest (RF) methodology. Results were compared to those of classical statistics.

**Results:**

RF analysis required no elaborate data pre-processing. The ranking of the variables with respect to classification impact (subject diseased, or not) was remarkably similar irrespective of the evaluation method used, leading to identical clinical interpretation.

**Conclusion:**

In metabolomic classification in adult patients with complex congenital heart disease, RF analysis as a one-step method delivers the most adequate results with minimum effort. RF may serve as an adjunct to traditional statistics also in this small but crucial-to-monitor patient group.

## Introduction

In biology and medicine, the metabolome, i.e., the concentration of metabolites in tissue, body fluids, excrements, exhaled gas, or other biomaterial, reflects the metabolic state of an organism (1). Modern to a high degree automated laboratory technology allows for the simultaneous determination of a vast multitude of metabolites from minute specimens at moderate costs in order to assess the metabolic state of, or clarify biochemical pathways in, an organism (“metabolomics”) (2). One approach to interpret the data is to assess both diseased subjects (“patients”) and healthy controls. Any difference in the metabolomic pattern between groups gives a hint at the metabolic pathways altered in the patient group. Mathematically speaking, the individual metabolomic pattern classifies the subject as either patient or control. Using traditional statistical approaches such as regression modeling, this study aims to rank various metabolites with respect to their classification impact and to identify those metabolites most promising to enlighten biochemical pathways and pathophysiology underlying the disease. The ultimate goal is to develop new concepts for diagnostics, monitoring, and therapy. Such concepts play an important role in cardiology, particularly with regard to the risk of the development of heart failure in the small but increasing group of adult patients with complex congenital heart disease (CHD). Classical statistical analysis of such data typically comprising hundreds of variables is a challenge, especially if the number of subjects to compare is low, if relations between dependent and independent variables are not linear, or if normal distributions or same variance are not met, and while classical statistics' strength is their power to evaluate the accuracy of correlations, they are less accurate for outcome prediction. With machine learning (ML) tools, which can be able to learn from the actual data to analyse instead of pre-programmed assumptions and instructions, there are alternatives for the evaluation of big data, especially allowing for outcome prediction.

Pattern recognition is a strength of artificial neural networks (3) that perform well as a classification tool. However, presenting to the user as “black box,” even from the underlying theory, it is difficult to identify those input variables contributing most to the classification task (3).

The random forest (RF) approach seems to be more promising (4–6). Following a sophisticated algorithm, a large number of individual decision trees with respect to the relationship between an input variable and classification result are generated. Then, each subject's metabolomic pattern is subjected in sequence to all the decision trees, internally noting each classification made. The majority vote of the trees is the class the subject is ultimately assigned to (7). In addition to subject classification, the algorithm delivers both the rank order of the several input variables' classification impact and their numerical classification impact score (8). Thus, it should be easy to identify the most promising metabolites deserving further (statistical) analysis and interpretation.

Machine learning algorithms, among the RFs, have been reported to offer an alternative approach to standard prognostic modeling, especially in the field of adult cardiology (9), e.g., for patients with heart failure for readmission prediction (10) (*n* = 977 patients, 472 input variables), or patients with angina for ischemia prediction (11) (*n* = 932 patients, 43 input variables). Data for the application of ML tools, including RF applied to the growing but a much smaller group of adult patients with CHD, are scarce. To date, the London group reported the only large study on ML (neural network, no RF) applied to >10,000 adult patients with various forms of CHD (<20% of them with complex CHD) (12), which showed that ML algorithms trained on large datasets are suitable to estimate prognosis and potentially guide therapy in adult patients with CHD. Chu et al. recently reported the benefit of ML tools, including RF on pregnant women with CHD, to successfully predict maternal or neonatal adverse events (13) (*n* = 318 patients; heterogeneous cardiac diagnoses, <20% of the patients with complex CHD; 22 input variables). For adult patients with complex CHD (*n* = 386, among them 40% with Fontan circulation), the very first report on the successful application of ML tools (recursive partitioning) to predict executive function (14) has been recently published. The group of adults with Fontan circulation, where the blood flows passively into the lungs and where the single ventricle pumps the blood into the system, is at the highest risk of all patients with CHD to develop organ dysfunction over time. Thus, this group of patients is crucial to focus on, and it is crucial to monitor and predict outcomes to accurately develop timely interventions. However, the major issue is that these patients are scarce and that uniform monitoring among centers is challenging, as is the application of standard (especially imaging) diagnostic tools, which are designed for a biventricular heart. Using a traditional regression approach (heatmap correlation), we recently reported distinct patterns of serum analytes as derived by metabolomics, taking into account more than hundreds of analytes (“big data”). The hope is, with this large amount of serum data, to ultimately find analyte patterns that early delineate adverse function, hemodynamic, or organ alterations. Taking a step back to the classification of Fontan patients vs. controls, we aim at applying RF, which has not been done before, and proving the RF's performance and feasibility on real metabolomic data. We hypothesized that for research on the metabolism of this small but crucial to scrutinize patient group, RF may present a simple adjunct, or even alternative, to classical statistics with respect to identifying the most relevant variables in a classification task and numerically estimating their relative impact.

## Materials and methods

Data from two recent metabolomic studies on 20 adult Fontan patients (univentricular palliation of an underlying complex CHD) and 20 healthy controls matched for age and sex (15, 16) were reanalysed. While in our previous analysis (15, 16), we used a heatmap correlation model to evaluate our hypothesis, in this study, we used RF methodology for classification (programme RF++ rel. 1.0) (17). We developed two training models. Input data were all available raw data from our recently published work, comprising 30 [amino acids (15)] and 110 [phospholipids (16)] metabolomic variables, respectively. Output (prediction) was the classification of patients as diseased (Fontan patient) vs. non-diseased (control proband). The parameter settings—number of variables used, number of candidate predictors randomly drawn for a split (mtry, candidate predictors that are randomly drawn for a split) (*n* = 6 for the amino acid model; *n* = 11 for the phospholipid model), and number of trees (*n* = 12,000 for both the amino acid and the phospholipid model)—were chosen as suggested by the software used (8, 17, 18). On each dataset, we performed several runs: for amino acids, we performed 9 runs: mtry, range 4–12 (default: 6); trees, range 1,000–40,000 (default: 12,000). For lipids, we performed 14 runs: mtry, range 6–15 (default: 11); trees, range 1,000–40,000 (default: 12,000) (Supplementary material 1).

All calculations were done using an ordinary notebook personal computer. The results were compared to those of classical statistics (15, 16). Finally, the method's feasibility was judged.

## Results

With respect to *amino acids and derivatives*, the RF run comprised all 30 metabolites analyzed by classical statistics (15). Subject classification (patient vs. control) was correct in 80% (accuracy score). The ranking of the variables with regard to classification impact given both as “permutation-based proportion” (8) and the slightly more expressive “mean decrease in the margin (MDM)” (19) was strikingly similar irrespective of the evaluation method used: the metabolites with an RF rank of 1–3 appeared among the topmost three metabolites as ranked by classical statistics (Table 1, top), leading to identical clinical interpretation (15).

**Table 1:** Random forest analysis.

**Random forest**		**Classical statistics** **(****15**, **16****)**		**Metabolite**
**Rank**	**Score**	**Rank**	**Fold change**	
AMS			
1	0.0751	2	1.78	Met-SO/Met
2	0.0461	3	1.64	Met-SO
3	0.0405	1	1.83	Glu
4	0.0342	7	1.26	ADMA
5	0.0171	5	1.35	t4-OH-Pro
6	0.0130	11	−1.19	His
7	0.0123	6	−1.32	Tau
8	0.0122	10	−1.21	Thr
9	0.0099	13	−1.16	Asn
10	0.0030	4	1.44	Alpha-AAA
**Lipids**
1	0.0459	2	−1.83	PC aa C34:4
2	0.0303	18	−1.38	SM (OH) C22:1
3	0.0294	13	−1.41	PC aa C32:3
4	0.0245	38	−1.30	SM C24:0
5	0.0171	67	−1.21	PC ae C38:4
6	0.0155	1	−1.92	PC aa C36:6
7	0.0143	24	−1.36	SM (OH) C24:1
8	0.0141	3	−1.75	PC aa C32:2
9	0.0128	51	−1.26	PC ae C32:1
10	0.0121	9	−1.44	PC ae C30:0
11	0.0112	5	−1.52	PC aa C30:0
12	0.0112	32	−1.31	PC ae C40:3
13	0.0085	33	−1.31	PC ae C40:4
14	0.0075	25	−1.35	SM C26:0
15	0.0068	72	−1.18	SM C24:1
16	0.0065	17	−1.38	PC ae C40:1
17	0.0063	10	−1.43	PC ae C42:4
18	0.0054	22	−1.36	PC ae C40:2
19	0.0048	23	−1,36	PC ae C44:4
20	0.0045	35	−1.31	SM (OH) C22:2

Specific serum metabolite and its classification impact compared to classical statistics. Only the topmost 10 (out of 30 for amino acids) and 20 (out of 110 for phospholipids) ranks are given. AMS, amino acids/derivatives (random forest parameter setting: no. of variables used, 30; the number of candidate predictors randomly drawn for a split (mtry), 6; no. of trees, 12,000). The first 10 ranks are given. Lipids, phospholipids (random forest parameter setting: no. of variables used, 110; mtry, 11; no. of trees, 12,000). The first 20 ranks are given. Random forest: rank, rank order; score, classification impact score (MDM, mean decrease in margin). Classical statistics: rank, rank order according to heatmap correlation analysis (15, 16); fold change, ratio between patients' and the controls' serum concentration of metabolites, a positive (negative) value indicating a higher (lower) metabolite concentration in patients. Metabolite, name of metabolite [AMS, Met-SO/Met, ratio of methionine sulfoxide/methionine; Met-SO, methionine sulfoxide; Glu, glutamic acid; ADMA, asymmetric dimethylarginine; t4-OH-Pro, trans-4-hydroxyproline; Tau, taurine; His, histidine; Thr, threonine; Asn, asparagine; Alpha-AAA, alpha aminoadipic acid. Lipids: PC, phosphatidylcholine; SM, sphingomyelin; aa, diacyl group; ae, acyl-alkyl group; x in Cx, number of carbon atoms in the diacyl/acyl-alkyl group; z in Cx:z, location of the double bond in the diacyl/acyl-alkyl group]. Note the rather small differences in the ranking scores in their respective fold changes from rank ≥5, indicating that the rank order of those less important metabolites inherits some uncertainty.

With respect to *phospholipids* (16), the RF run simultaneously comprising all 110 metabolites that had been subjected to classical statistics resulted in a correct subject classification of 85%. Compared to classical statistics, RF analysis resulted in a similar ranking of the top variables with regard to classification impact: the RF ranks 1–8 comprised the topmost 3 metabolites ranked by classical statistics (Table 1, bottom), again suggesting identical clinical interpretation (16).

**Figure 1:**
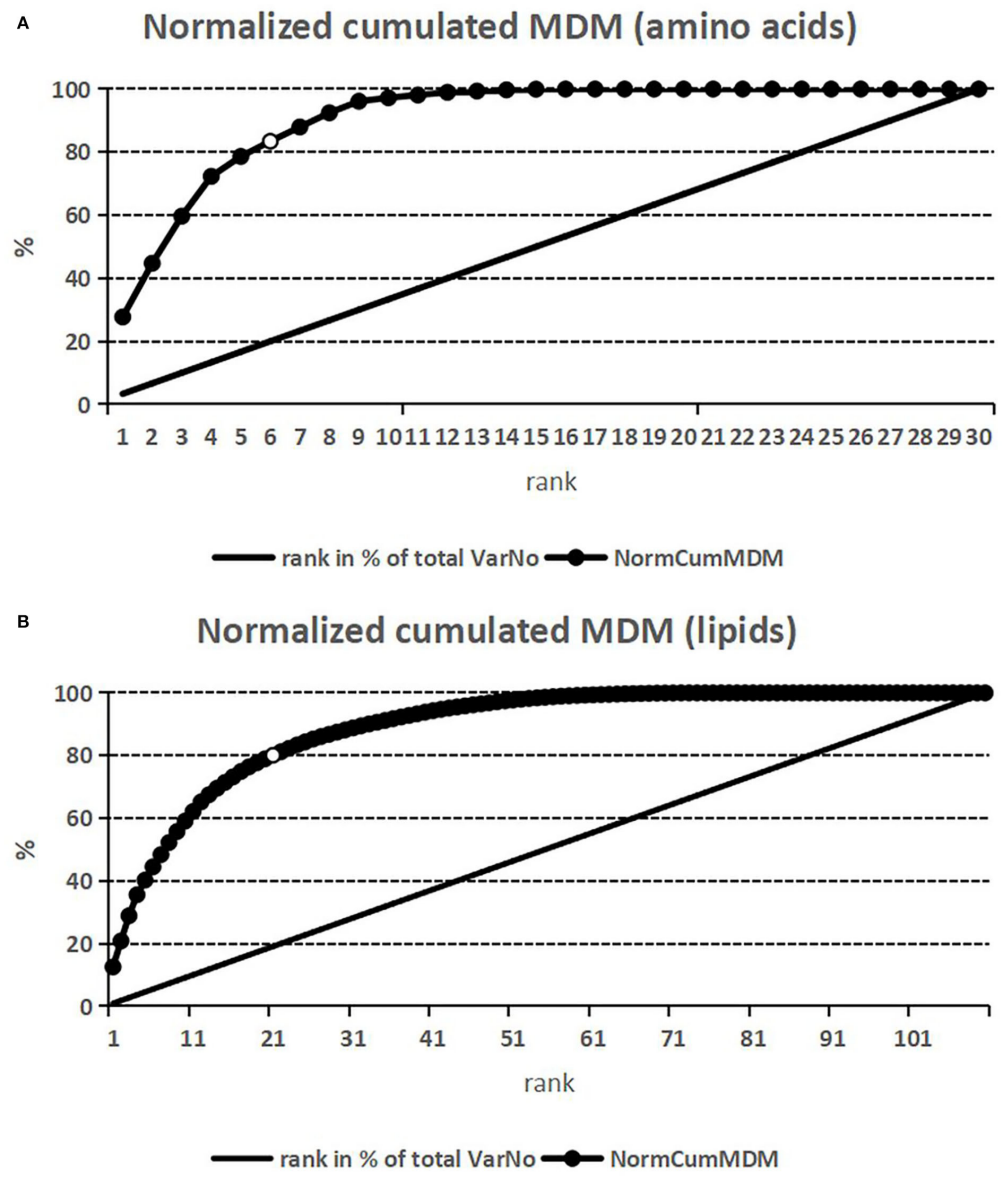
Cumulative classification impact (diseased vs. non-diseased)—normalized to 100%—of the respective metabolites (dotted curves) vs. absolute impact rank. The linear line indicates the analyte's rank normalized to 100%. %, percentage; MDM, mean decrease in the margin (variable importance with respect to classification into diseased vs. non-diseased); NormCumMDM, normalized cumulated mean decrease in the margin; rank, rank with respect to classification impact MDM, starting with the highest impact as rank 1. **(A)** *Amino acids*. The topmost ranked 6/30 amino acids (20% of analytes; open circle) account for 80% of the classification total. **(B)** *Phospholipids*. The topmost ranked 21/110 phospholipids (19% of analytes; open circle) account for 80% of the classification total.

The *cumulative impact* of amino acids and phospholipids is shown in Figures 1A,B. With a cumulative impact normalized to 100% (per group), it becomes obvious that already a small number of analytes (the topmost ranked 6/30 as for amino acids and the topmost ranked 21/110 as for phospholipids) account for 80% of the classification optimum.

Doing repeated RF runs, all results proved robust with regard to even wide variations in the RF parameter setting (Supplementary material 1). Each run takes only a few seconds to complete.

Classical statistics required elaborate pre-processing of the data (logspline imputing of values < limit of detection; log2 transformation/normalization; outlier detection and elimination), and p-correction for multiple testing thus losing sensitivity (15, 16). No such data pre-processing was required for the RF analyses (8). While classical statistics required the participation of an expert statistician as a prerequisite for dependable results and sound interpretation, there was no such requirement with respect to RF analysis.

## Discussion

Machine learning algorithms have been reported to successfully estimate prognosis and guide therapy for adult patients with various CHD (9–14). As we have shown, also in patients with complex CHD and Fontan circulation, where patient numbers are low and input variable numbers may be large, both ML algorithms (RF analysis in our case) and classical statistics are potent classification tools. With respect to amino acids (phospholipids), the RF topmost ranking 6 (21) metabolites inherit more than 80% of the classification competence. Both methods depict a heavily overlapping (especially true for amino acids, functioning more heterogeneously than phospholipids) albeit not identical bunch of metabolites as most promising for classification (diseased vs. non-diseased), deserving further analysis and interpretation. Thus, the clinical conclusions to be drawn (15, 16) are identical. The topmost ranked metabolites—ranked by either RF or classical statistics—(Met-SO/Met, Met-SO, Glu; phospholipids with medium and long alkyl chains) suggest that in Fontan patients, the signaling and inflammatory pathways hinting at oxidative stress and endothelial dysfunction, as well as energy and structural metabolism are affected, as reported for other cardiovascular circumstances as for patients with heart failure (15, 16, 20, 21). The metabolites ranking ≥5 do not show but small differences in their absolute fold change values between successive ranks; beyond rank 20—particularly with the lipids (as with a set of proteomics data, see performance analysis as mentioned under limitations)—the differences are minimal, rendering further ranking meaningless for clinical interpretation (biological variability, small sample size).

By no means, classical heatmap correlation analysis is an objective method. Actually, its results to some degree depend on the expert operator's parameter setting according to clinical judgement (22). Thus, it must not be regarded as the golden standard for the analysis of such types of data as given here. It might well be that RF analysis reveals interdependencies ignored by traditional regression analysis and *vice versa*. Hence, both types of analysis are complementing each other.

Although RF analysis is technically much easier to perform than sophisticated classical statistics, it delivers dependable results even to the statistically less experienced. RF parameter settings are uncritical, even if the parameters are well beyond the range suggested by the programme used. Contrary to classical statistics, RF can handle huge numbers of variables per case without performance loss (8). Its output is easy to interpret. While each variable's classification impact is numerically scored as “permutation-based proportion” and “mean decrease in margin (MDM)” (8, 19), classical indicators of the grade of the significance of the various results—equivalent to eta^2^ and *p*-value in classical statistics or the here used fold change—are missing. If this is of importance, it is debatable as long as the overall interpretation of the results is the same. Insofar, metabolomic RF analysis should be considered an alternative to classical statistics. In case one feels uneasy about the lack of such measures of determination and prefers classical statistics, in the first step one could perform RF analysis as an adjunct in order to identify the most promising variables, and in the next step subject a set of selected variables to classical statistics thus reducing processing effort.

### Limitations, confounders

The analysis presented in this study depends on data of only 40 subjects, hence one should be careful with generalizing our findings on methodology. Independent internal validation would have been desirable for both RF and classical statistics. Two features might, however, compensate to some degree for this missing internal validation: For RF, we used out-of-bag (OOB) prediction, meaning that every tree in the RF results from only a fraction of the data points in the dataset. OOB predictions with respect to a specific variable are generated by using only those trees that did *not* use that variable to generate the predictions. This provides a more unbiased estimate of the prediction error of the random forest and should give a similar error rate to that when making predictions on a new and independent dataset. Still, while the OOB error rate in RF is quite acceptable, due to the lack of an independent validation sample for classical statistics, an error rate cannot be provided, thus precluding the comparison of the two methods in this regard.

For example, in another performance estimate on the same 40 subjects and a set of 526 proteomics-derived metabolites per subject (work submitted), RF analysis tagged 4 of the 6 most promising metabolites according to classical statistics as most important (the odds of such a hit by guessing are <1 in a million), underlining the potential of RF. As for the cumulative impact, the topmost ranked 27/526 analytes accounted for 80% of the classification total.

With RF, we used easy-to-operate software freely available online and fully meeting our demands (8, 17). But, in other settings, more sophisticated RF software might be more adequate (23).

## Conclusion

Our preliminary data on a small patient group implies that RF is applicable to the scarce but crucial to monitor group of patients with complex diseases which impact metabolism. In a metabolomic classification setting, RF analysis as a one-step method delivers the most adequate results with a minimum of effort. RF analysis is both a compelling stand-alone tool and an adjunct to classical statistics. These conclusions have to be proven by analysis of a larger patient number.

## Study registration number

Study no. 20210803-2653 (registered at the Competence Center for Clinical Trials, Medical University of Innsbruck).

## Data availability statement

The original contributions presented in the study are included in the article/Supplementary material, further inquiries can be directed to the corresponding authors. This study is a subwork of the main study protocol (Trial registration number: ClinicalTrials.gov Identifier NCT03886935).

## Ethics statement

The studies involving human participants were reviewed and approved by Local Ethics Committee of the Medical University of Innsbruck, Austria (AN2015-0303 357/4.3 4507a) and of the Heart and Diabetes Center North Rhine-Westphalia, Ruhr-University of Bochum, Germany (AZ 53/2016). The methods were carried out in accordance with the Helsinki Declaration and the International Conference on Harmonization Good Clinical Practice Guidelines. The patients/participants provided their written informed consent to participate in this study.

## Author contributions

Conceptualization, validation, data curation, writing—original draft preparation, supervision, and project administration: MM and EM. Methodology, formal analysis, and visualization: EM. Investigation and writing—review and editing: MM, EM, KL, K-OD, and SS-B. Resources and funding acquisition: MM. All authors have read and agreed to the published version of the manuscript.

## Funding

MM received funding from the Austrian Science Fund (no. KLI1036-B), the Austrian Society of Pediatrics (no. 04/2021), the Tiroler Wissenschaftsförderung (no. UNI-0404-2126), and the Medizinischer Forschungsfonds Tirol (no. 327). The funders had no role in the design of the study; in the collection, analyses, or interpretation of data; in the writing of the manuscript; or in the decision to publish the results.

## Conflict of interest

The authors declare that the research was conducted in the absence of any commercial or financial relationships that could be construed as a potential conflict of interest.

## Publisher's note

All claims expressed in this article are solely those of the authors and do not necessarily represent those of their affiliated organizations, or those of the publisher, the editors and the reviewers. Any product that may be evaluated in this article, or claim that may be made by its manufacturer, is not guaranteed or endorsed by the publisher.
