# Supplementary material for: Metabolomics and random forests in patients with complex congenital heart disease
Source: Front Cardiovasc Med. 2022 Oct 5;9:994068. doi: 10.3389/fcvm.2022.994068 (PMC9581308; doi:10.3389/fcvm.2022.994068)
Supplement: Supplementary file 1 [file Data_Sheet_1.PDF]

Amino acids

Mtry=6 Trees=12000 Seed=9 (as selected for analysis described in the manuscript)

Sample-level *Error Rate*: 0.2

Sample-level Confusion Matrix: (rows = predicted, cols = actual)

15	3
5	17

Variable importance:

Permutation-based Proportion		Mean Decrease in Margin (**MDM**)	
ID	Score	ID	Score
30	0.0480	30	0.0751
24	0.0296	24	0.0461
7	0.0262	7	0.0405
22	0.0219	22	0.0342
28	0.0109	28	0.0171
9	0.0082	9	0.0130
29	0.0081	29	0.0123
18	0.0080	18	0.0122
3	0.0064	3	0.0099
27	0.0018	23	0.0030
23	0.0016	27	0.0024
12	0.0015	12	0.0023
14	0.0008	14	0.0011
17	0.0005	17	0.0010
1	0.0005	1	0.0006
26	0.0002	6	0.0001
6	0.0001	26	0.0000
10	-0.0001	10	-0.0000
11	-0.0001	19	-0.0003
20	-0.0002	11	-0.0005
19	-0.0002	20	-0.0005
2	-0.0004	2	-0.0007
21	-0.0005	21	-0.0009
13	-0.0010	4	-0.0017
4	-0.0010	13	-0.0018
16	-0.0012	16	-0.0019
8	-0.0012	8	-0.0020
15	-0.0013	15	-0.0020
5	-0.0016	25	-0.0026
25	-0.0017	5	-0.0027

Mtry=6 Trees=**1000** Seed=9

Sample-level Error Rate: 0.225

Sample-level Confusion Matrix: (rows = predicted, cols = actual)

15	4
5	16

Variable importance:

Permutation-based Proportion		Mean Decrease in Margin (**MDM**)	
ID	Score	ID	Score
30	0.0453	30	0.0705
24	0.0273	24	0.0415
7	0.0247	7	0.0400
22	0.0185	22	0.0282
29	0.0138	29	0.0213
28	0.0114	28	0.0181
18	0.0097	18	0.0153
9	0.0060	9	0.0098
3	0.0055	3	0.0068
27	0.0022	27	0.0029
23	0.0014	23	0.0024
12	0.0013	26	0.0021
26	0.0009	12	0.0019
10	0.0009	14	0.0017
14	0.0009	10	0.0013
11	0.0008	17	0.0009
1	0.0007	1	0.0009
17	0.0006	11	0.0009
19	-0.0002	5	-0.0006

```
     5   -0.0002          19   -0.0006
    13   -0.0003          13   -0.0009
     2   -0.0006           6   -0.0011
    20   -0.0007           2   -0.0015
     6   -0.0008          20   -0.0016
     4   -0.0010           8   -0.0019
     8   -0.0015           4   -0.0019
    16   -0.0016          15   -0.0023
    25   -0.0017          25   -0.0026
    15   -0.0018          16   -0.0027
    21   -0.0019          21   -0.0030
```

Mtry=6 Trees=**6000** Seed=9

```
Sample-level Error Rate: 0.2
Sample-level Confusion Matrix: (rows = predicted, cols = actual)
    15     3
     5    17

Variable importance:
Permutation-based   |   Mean Decrease in
Proportion          |   Margin (MDM)
    ID    Score     |       ID    Score
    30    0.0469          30    0.0737
    24    0.0300          24    0.0468
     7    0.0254           7    0.0403
    22    0.0220          22    0.0346
    28    0.0117          28    0.0182
     9    0.0090           9    0.0138
    29    0.0086          29    0.0134
    18    0.0079          18    0.0116
     3    0.0060           3    0.0091
    27    0.0019          27    0.0027
    12    0.0012          23    0.0019
    23    0.0010          12    0.0016
     1    0.0009           1    0.0012
    26    0.0007          17    0.0008
    17    0.0005          26    0.0008
    14    0.0004          14    0.0006
    11    0.0003          11    0.0003
     6    0.0002           6    0.0002
    10   -0.0001          10   -0.0002
     2   -0.0002          19   -0.0003
    19   -0.0002           2   -0.0004
    21   -0.0005          21   -0.0007
    20   -0.0007          20   -0.0012
    16   -0.0008          16   -0.0012
    13   -0.0010          13   -0.0017
     4   -0.0011          15   -0.0019
    15   -0.0012           4   -0.0019
     5   -0.0015           5   -0.0024
     8   -0.0017           8   -0.0028
    25   -0.0021          25   -0.0030
```

Mtry=6 Trees=**20000** Seed=9

```
Sample-level Error Rate: 0.2
Sample-level Confusion Matrix: (rows = predicted, cols = actual)
    15     3
     5    17

Variable importance:
Permutation-based   |   Mean Decrease in
Proportion          |   Margin (MDM)
    ID    Score     |       ID    Score
    30    0.0474          30    0.0742
    24    0.0298          24    0.0463
     7    0.0262           7    0.0412
    22    0.0226          22    0.0352
    28    0.0106          28    0.0166
    29    0.0090           9    0.0140
     9    0.0089          29    0.0134
    18    0.0078          18    0.0120
     3    0.0064           3    0.0098
```

```
    27    0.0020          23    0.0028
    23    0.0017          27    0.0027
    12    0.0016          12    0.0027
     1    0.0008           1    0.0011
    26    0.0008          17    0.0009
    14    0.0006          26    0.0009
    17    0.0006          14    0.0009
     6    0.0002           6    0.0002
    11    0.0002          11    0.0001
    10   -0.0001          10   -0.0001
    19   -0.0002          19   -0.0005
     2   -0.0002           2   -0.0005
    21   -0.0006          21   -0.0010
    20   -0.0007          20   -0.0012
    13   -0.0008          13   -0.0014
     4   -0.0011          16   -0.0018
    16   -0.0011           4   -0.0020
     8   -0.0012          15   -0.0020
    15   -0.0012           8   -0.0021
    25   -0.0017          25   -0.0027
     5   -0.0019           5   -0.0030
```

Mtry=6 Trees=**40000** Seed=9

```
Sample-level Error Rate: 0.2
Sample-level Confusion Matrix: (rows = predicted, cols = actual)
    15     3
     5    17
```

Variable importance:

```
Permutation-based   |   Mean Decrease in
Proportion          |   Margin (MDM)
    ID    Score     |       ID    Score
    30    0.0469          30    0.0730
    24    0.0293          24    0.0458
     7    0.0258           7    0.0404
    22    0.0232          22    0.0362
    28    0.0108          28    0.0170
    29    0.0085          29    0.0128
     9    0.0083           9    0.0127
    18    0.0080          18    0.0122
     3    0.0065           3    0.0099
    27    0.0022          27    0.0032
    23    0.0019          23    0.0031
    12    0.0015          12    0.0022
     1    0.0010           1    0.0015
    26    0.0010          26    0.0013
    14    0.0007          14    0.0010
    17    0.0005          17    0.0008
     6    0.0005           6    0.0006
    11   -0.0000          11   -0.0003
    10   -0.0003          10   -0.0005
    19   -0.0003          19   -0.0005
     2   -0.0003           2   -0.0006
    20   -0.0004          20   -0.0008
    21   -0.0006          21   -0.0008
    13   -0.0007          13   -0.0011
     4   -0.0010           4   -0.0016
    16   -0.0011          15   -0.0017
    15   -0.0011          16   -0.0018
     8   -0.0013           8   -0.0022
     5   -0.0019          25   -0.0029
    25   -0.0019           5   -0.0031
```

Mtry=**4** Trees=12000 Seed=9

```
Sample-level Error Rate: 0.225
Sample-level Confusion Matrix: (rows = predicted, cols = actual)
    15     4
     5    16
```

Variable importance:

```
Permutation-based   |   Mean Decrease in
Proportion          |   Margin (MDM)
    ID    Score     |       ID    Score
```

```
    30    0.0382          30    0.0599
    24    0.0266          24    0.0417
     7    0.0242           7    0.0380
    22    0.0205          22    0.0316
    28    0.0123          28    0.0192
    29    0.0094          29    0.0141
     9    0.0083           9    0.0127
    18    0.0080          18    0.0120
     3    0.0072           3    0.0109
    23    0.0020          23    0.0033
    12    0.0019          12    0.0029
    27    0.0018          27    0.0028
     6    0.0010           6    0.0017
     1    0.0009           1    0.0014
    26    0.0006          26    0.0008
    14    0.0002          17    0.0002
    17    0.0000          14    0.0001
     2   -0.0000          13   -0.0000
    13   -0.0001           2   -0.0003
    11   -0.0003          20   -0.0006
    10   -0.0004          11   -0.0007
    20   -0.0004          10   -0.0007
    21   -0.0007          21   -0.0009
    19   -0.0007          19   -0.0014
     8   -0.0010           8   -0.0015
    15   -0.0012          15   -0.0020
    16   -0.0015          16   -0.0025
     4   -0.0018           4   -0.0030
     5   -0.0019           5   -0.0030
    25   -0.0023          25   -0.0035
```

Mtry=**8** Trees=12000 Seed=9

```
Sample-level Error Rate: 0.225
Sample-level Confusion Matrix: (rows = predicted, cols = actual)
    15     4
     5    16

Variable importance:
Permutation-based   |   Mean Decrease in
Proportion          |   Margin (MDM)
    ID    Score     |       ID    Score
    30    0.0544          30    0.0850
    24    0.0309          24    0.0488
     7    0.0250           7    0.0393
    22    0.0229          22    0.0358
    28    0.0102          28    0.0159
    29    0.0091          29    0.0137
     9    0.0083           9    0.0129
    18    0.0080          18    0.0119
     3    0.0062           3    0.0093
    27    0.0019          27    0.0027
    12    0.0013          12    0.0021
    23    0.0013          23    0.0019
     1    0.0012           1    0.0018
    26    0.0010          26    0.0015
    14    0.0009          14    0.0012
    17    0.0003          17    0.0006
    11    0.0001           6    0.0002
     6    0.0001          11    0.0002
    19   -0.0003          19   -0.0002
    10   -0.0004          10   -0.0007
     2   -0.0004          21   -0.0007
    21   -0.0005           2   -0.0008
    20   -0.0007          20   -0.0012
    13   -0.0009          13   -0.0015
    15   -0.0012          15   -0.0019
     4   -0.0013           4   -0.0023
     8   -0.0015          16   -0.0024
    16   -0.0015           8   -0.0024
    25   -0.0018          25   -0.0028
     5   -0.0018           5   -0.0030
```

Mtry=**12** Trees=12000 Seed=9

Sample-level Error Rate: 0.225

Sample-level Confusion Matrix: (rows = predicted, cols = actual)

15	4
5	16

Variable importance:

Permutation-based Proportion		Mean Decrease in Margin (**MDM**)	
ID	Score	ID	Score
30	0.0625	30	0.0975
24	0.0298	24	0.0472
7	0.0256	7	0.0401
22	0.0224	22	0.0351
9	0.0083	9	0.0128
28	0.0076	28	0.0117
18	0.0073	18	0.0106
29	0.0072	29	0.0106
3	0.0047	3	0.0069
27	0.0024	27	0.0038
23	0.0017	23	0.0027
26	0.0012	26	0.0017
1	0.0009	1	0.0013
17	0.0009	17	0.0011
12	0.0006	12	0.0009
14	0.0006	14	0.0008
19	0.0000	19	0.0001
2	-0.0001	2	-0.0002
11	-0.0002	10	-0.0005
10	-0.0003	11	-0.0006
21	-0.0003	21	-0.0006
15	-0.0006	15	-0.0009
20	-0.0007	20	-0.0013
8	-0.0008	8	-0.0013
4	-0.0008	4	-0.0015
6	-0.0009	13	-0.0017
13	-0.0010	6	-0.0017
16	-0.0012	16	-0.0021
5	-0.0012	5	-0.0021
25	-0.0018	25	-0.0030

Mtry=**12** Trees=**20000** Seed=9

Sample-level Error Rate: 0.225

Sample-level Confusion Matrix: (rows = predicted, cols = actual)

15	4
5	16

Variable importance:

Permutation-based Proportion		Mean Decrease in Margin (**MDM**)	
ID	Score	ID	Score
30	0.0634	30	0.0992
24	0.0296	24	0.0468
7	0.0256	7	0.0401
22	0.0228	22	0.0357
9	0.0092	9	0.0139
28	0.0078	28	0.0121
29	0.0075	29	0.0113
18	0.0073	18	0.0112
3	0.0042	3	0.0062
27	0.0022	27	0.0032
23	0.0014	23	0.0024
26	0.0014	26	0.0021
17	0.0012	17	0.0019
12	0.0011	12	0.0017
14	0.0009	14	0.0013
1	0.0007	1	0.0010
2	0.0002	2	0.0004
19	-0.0002	19	-0.0003
21	-0.0003	21	-0.0004
11	-0.0003	11	-0.0005
10	-0.0004	10	-0.0006
20	-0.0006	20	-0.0010
6	-0.0007	4	-0.0012
4	-0.0007	6	-0.0012

15	-0.0008	15	-0.0013
13	-0.0009	13	-0.0015
8	-0.0010	8	-0.0016
16	-0.0012	16	-0.0020
5	-0.0015	5	-0.0026
25	-0.0018	25	-0.0027

Phospholipids

Mtry=**11** Trees=**12000** Seed=9 **(as selected fort he analysis described in the manuscript)**

Sample-level Error Rate: 0.15
Sample-level Confusion Matrix: (rows = predicted, cols = actual)

18	4
2	16

Variable importance:

Permutation-based Proportion		Mean Decrease in Margin (**MDM**)	
ID	Score	ID	Score
36	0.0284	36	0.0459
99	0.0188	99	0.0303
32	0.0181	32	0.0294
107	0.0155	107	0.0245
79	0.0104	79	0.0171
43	0.0096	43	0.0155
101	0.0089	101	0.0143
31	0.0087	31	0.0141
63	0.0076	63	0.0128
61	0.0074	61	0.0121
84	0.0069	28	0.0112
28	0.0067	84	0.0112
85	0.0051	85	0.0085
109	0.0049	109	0.0075
108	0.0040	108	0.0068
82	0.0038	82	0.0065
91	0.0038	91	0.0063
83	0.0032	83	0.0054
94	0.0029	94	0.0048
100	0.0028	100	0.0045
89	0.0026	12	0.0042
90	0.0026	90	0.0041
12	0.0025	89	0.0040
49	0.0025	49	0.0039
102	0.0021	104	0.0035
104	0.0021	102	0.0034
13	0.0019	13	0.0029
67	0.0017	68	0.0026
68	0.0016	67	0.0026
87	0.0016	87	0.0025
110	0.0015	86	0.0024
66	0.0014	66	0.0024
86	0.0014	110	0.0022
92	0.0013	78	0.0021
78	0.0012	65	0.0020
97	0.0012	16	0.0020
65	0.0012	92	0.0020
71	0.0012	98	0.0019
16	0.0012	97	0.0019
98	0.0012	27	0.0019
27	0.0012	71	0.0017
42	0.0009	42	0.0016
55	0.0009	55	0.0015
75	0.0008	20	0.0013
35	0.0008	37	0.0012
20	0.0007	64	0.0012
64	0.0007	75	0.0012
96	0.0007	35	0.0012
37	0.0007	74	0.0012
74	0.0007	96	0.0011
72	0.0007	72	0.0010
3	0.0006	3	0.0009
21	0.0005	21	0.0009
4	0.0004	56	0.0007
1	0.0004	1	0.0007
56	0.0004	45	0.0006
62	0.0004	4	0.0006
45	0.0004	47	0.0005
47	0.0003	70	0.0004
70	0.0003	62	0.0004
93	0.0002	93	0.0003
77	0.0002	95	0.0003
95	0.0002	77	0.0003

```
     5    0.0001          81    0.0003
    30    0.0001          57    0.0003
    81    0.0001          11    0.0002
    57    0.0001          15    0.0002
    73    0.0001           5    0.0002
    15    0.0001          30    0.0002
    11    0.0001          41    0.0001
    46    0.0001          19    0.0001
    19    0.0001          73    0.0001
     6    0.0000          46    0.0000
    41    0.0000           6   -0.0000
     8   -0.0000          17   -0.0000
    17   -0.0000           8   -0.0001
    76   -0.0001           2   -0.0002
    52   -0.0001          76   -0.0002
    38   -0.0001          38   -0.0002
    23   -0.0001          52   -0.0002
     2   -0.0001          23   -0.0002
    18   -0.0001          48   -0.0002
     7   -0.0001          18   -0.0003
    48   -0.0001          25   -0.0003
    58   -0.0002           7   -0.0003
    25   -0.0002          58   -0.0003
    26   -0.0002          88   -0.0003
    88   -0.0002          26   -0.0003
    60   -0.0002          60   -0.0003
    69   -0.0002          80   -0.0004
    80   -0.0002         103   -0.0004
    40   -0.0002          40   -0.0004
   103   -0.0003          69   -0.0004
    14   -0.0003          14   -0.0004
    22   -0.0003          22   -0.0005
    50   -0.0003         105   -0.0005
    53   -0.0003          50   -0.0005
   105   -0.0003          53   -0.0005
    34   -0.0003          33   -0.0005
    51   -0.0003          34   -0.0005
    33   -0.0003          54   -0.0006
    54   -0.0003          51   -0.0006
    39   -0.0004          39   -0.0006
   106   -0.0004         106   -0.0006
    24   -0.0004          24   -0.0007
    44   -0.0004          44   -0.0007
    29   -0.0005          29   -0.0008
    10   -0.0005           9   -0.0008
    59   -0.0005          59   -0.0009
     9   -0.0005          10   -0.0009
```

Mtry=11 Trees=**1000** Seed=9

```
Sample-level Error Rate: 0.15
Sample-level Confusion Matrix: (rows = predicted, cols = actual)
    18     4
     2    16
```

Variable importance:

```
Permutation-based   |   Mean Decrease in
Proportion          |   Margin (MDM)
    ID    Score     |       ID    Score
    36    0.0266          36    0.0419
    32    0.0195          32    0.0320
    99    0.0169          99    0.0272
   107    0.0137         107    0.0215
   101    0.0098         101    0.0163
    43    0.0091          79    0.0149
    79    0.0088          43    0.0140
    28    0.0081          28    0.0138
    31    0.0080          31    0.0128
    63    0.0067          63    0.0113
   109    0.0064          61    0.0101
    61    0.0061         109    0.0099
    85    0.0058          85    0.0096
    84    0.0048          84    0.0079
    91    0.0045          91    0.0072
    82    0.0043          82    0.0070
```

94	0.0036	94	0.0063
83	0.0034	83	0.0062
90	0.0032	90	0.0051
108	0.0030	108	0.0049
100	0.0030	12	0.0048
102	0.0029	89	0.0047
89	0.0029	100	0.0045
12	0.0027	68	0.0045
49	0.0024	102	0.0043
68	0.0024	78	0.0042
67	0.0022	49	0.0036
78	0.0022	13	0.0036
13	0.0021	104	0.0033
104	0.0019	67	0.0028
27	0.0018	86	0.0027
92	0.0016	27	0.0026
86	0.0015	110	0.0022
87	0.0013	92	0.0022
110	0.0013	87	0.0022
42	0.0012	55	0.0019
20	0.0012	20	0.0019
71	0.0011	42	0.0018
62	0.0010	71	0.0018
47	0.0010	4	0.0017
55	0.0010	37	0.0017
65	0.0009	47	0.0015
4	0.0009	74	0.0015
74	0.0008	98	0.0014
3	0.0008	65	0.0013
98	0.0008	62	0.0013
37	0.0008	75	0.0012
96	0.0007	3	0.0011
1	0.0007	96	0.0010
75	0.0006	1	0.0010
16	0.0006	46	0.0009
2	0.0006	16	0.0009
45	0.0006	19	0.0009
19	0.0005	45	0.0009
46	0.0005	72	0.0008
70	0.0005	66	0.0008
66	0.0005	35	0.0007
72	0.0004	2	0.0006
35	0.0004	70	0.0005
56	0.0003	57	0.0004
76	0.0002	76	0.0004
73	0.0002	56	0.0004
11	0.0001	77	0.0002
57	0.0001	73	0.0002
95	0.0001	21	0.0002
14	0.0000	11	0.0001
88	0.0000	53	0.0000
26	0.0000	97	0.0000
97	-0.0000	88	-0.0000
34	-0.0000	14	-0.0000
81	-0.0000	7	-0.0001
21	-0.0000	34	-0.0001
77	-0.0000	95	-0.0001
52	-0.0001	103	-0.0001
7	-0.0001	52	-0.0001
48	-0.0001	81	-0.0001
53	-0.0001	48	-0.0002
60	-0.0001	33	-0.0002
103	-0.0001	23	-0.0003
8	-0.0002	58	-0.0003
15	-0.0002	17	-0.0003
58	-0.0002	26	-0.0003
22	-0.0002	18	-0.0003
33	-0.0002	22	-0.0004
23	-0.0002	8	-0.0004
17	-0.0002	60	-0.0004
10	-0.0002	25	-0.0004
18	-0.0002	10	-0.0005
25	-0.0003	39	-0.0005
105	-0.0003	105	-0.0005
41	-0.0003	15	-0.0006
93	-0.0003	41	-0.0006
51	-0.0003	30	-0.0006

```
    39   -0.0003          54   -0.0006
    30   -0.0004          93   -0.0007
    54   -0.0004          64   -0.0007
    64   -0.0004           5   -0.0008
     6   -0.0004          51   -0.0008
     5   -0.0005           6   -0.0009
    69   -0.0005          69   -0.0010
    80   -0.0006          80   -0.0011
    40   -0.0006          38   -0.0011
     9   -0.0007           9   -0.0011
    29   -0.0007          40   -0.0011
    50   -0.0007          50   -0.0012
    38   -0.0008          24   -0.0013
    59   -0.0008          44   -0.0013
    24   -0.0008          59   -0.0014
    44   -0.0009          29   -0.0014
   106   -0.0010         106   -0.0016
```

Mtry=11 Trees=4**000** Seed=9

```
Sample-level Error Rate: 0.15
Sample-level Confusion Matrix: (rows = predicted, cols = actual)
    18     4
     2    16
```

Variable importance:

```
Permutation-based   |   Mean Decrease in
Proportion          |   Margin (MDM)
    ID    Score     |       ID    Score
    36    0.0290          36    0.0463
    32    0.0178          32    0.0293
    99    0.0172          99    0.0279
   107    0.0143         107    0.0224
    79    0.0105          79    0.0173
    43    0.0103          43    0.0163
   101    0.0095         101    0.0153
    31    0.0084          31    0.0139
    61    0.0074          63    0.0121
    63    0.0072          61    0.0117
    28    0.0068          28    0.0112
    84    0.0061          84    0.0101
   109    0.0054         109    0.0084
    85    0.0045          85    0.0073
   108    0.0039         108    0.0067
    82    0.0038          91    0.0062
    94    0.0036          82    0.0061
    91    0.0036          94    0.0059
    90    0.0034         100    0.0054
   100    0.0034          90    0.0052
    89    0.0028          83    0.0047
    83    0.0026          89    0.0045
    12    0.0025          12    0.0042
   104    0.0023         104    0.0038
    68    0.0018          65    0.0030
    92    0.0017          68    0.0030
    65    0.0017          78    0.0029
    67    0.0016          92    0.0026
    78    0.0015          86    0.0024
    49    0.0015          67    0.0024
   102    0.0014          98    0.0023
    86    0.0014          16    0.0022
    27    0.0014          27    0.0022
    13    0.0014          13    0.0022
    98    0.0014          49    0.0021
    16    0.0013         102    0.0021
    97    0.0011          97    0.0017
   110    0.0010          42    0.0016
    87    0.0009          66    0.0016
    66    0.0009         110    0.0016
    42    0.0009          37    0.0016
    74    0.0008          87    0.0015
    75    0.0008          75    0.0014
     1    0.0008          74    0.0014
    37    0.0008           1    0.0013
    96    0.0007          55    0.0012
```

71	0.0007	20	0.0011
62	0.0007	64	0.0011
56	0.0007	96	0.0010
64	0.0006	45	0.0010
20	0.0006	35	0.0010
55	0.0006	71	0.0010
45	0.0006	56	0.0009
35	0.0006	72	0.0009
3	0.0006	62	0.0009
4	0.0005	4	0.0008
72	0.0005	77	0.0008
70	0.0004	3	0.0007
57	0.0004	57	0.0007
77	0.0004	81	0.0006
81	0.0004	70	0.0005
47	0.0003	47	0.0004
46	0.0002	17	0.0003
17	0.0002	46	0.0003
30	0.0002	30	0.0003
93	0.0001	73	0.0003
73	0.0001	19	0.0002
21	0.0001	93	0.0002
19	0.0001	58	0.0001
58	0.0001	21	0.0001
11	0.0000	11	0.0000
18	-0.0000	95	-0.0000
95	-0.0000	23	-0.0001
15	-0.0001	8	-0.0001
7	-0.0001	7	-0.0001
88	-0.0001	52	-0.0001
8	-0.0001	76	-0.0001
23	-0.0001	2	-0.0001
2	-0.0001	18	-0.0001
52	-0.0001	41	-0.0002
76	-0.0001	88	-0.0002
6	-0.0001	60	-0.0002
60	-0.0001	38	-0.0003
38	-0.0002	15	-0.0003
22	-0.0002	103	-0.0003
5	-0.0002	80	-0.0003
80	-0.0002	22	-0.0003
41	-0.0002	6	-0.0003
26	-0.0002	39	-0.0003
48	-0.0002	105	-0.0004
39	-0.0002	25	-0.0004
105	-0.0002	48	-0.0004
40	-0.0002	33	-0.0005
25	-0.0003	40	-0.0005
33	-0.0003	26	-0.0005
103	-0.0003	5	-0.0005
53	-0.0003	34	-0.0005
34	-0.0004	53	-0.0006
51	-0.0004	51	-0.0007
14	-0.0004	44	-0.0007
69	-0.0004	50	-0.0007
59	-0.0005	14	-0.0008
50	-0.0005	69	-0.0008
24	-0.0005	59	-0.0008
106	-0.0005	24	-0.0008
44	-0.0005	106	-0.0009
9	-0.0005	9	-0.0010
29	-0.0006	29	-0.0010
54	-0.0006	54	-0.0010
10	-0.0007	10	-0.0011

Mtry=11 Trees=**20000** Seed=9

Sample-level Error Rate: 0.15

Sample-level Confusion Matrix: (rows = predicted, cols = actual)

18	4
2	16

Variable importance:

Permutation-based Proportion		Mean Decrease in Margin (**MDM**)	
ID	Score	ID	Score

36	0.0280	36	0.0452
99	0.0185	99	0.0297
32	0.0178	32	0.0291
107	0.0146	107	0.0233
79	0.0107	79	0.0175
43	0.0094	43	0.0150
101	0.0092	101	0.0149
31	0.0086	31	0.0138
61	0.0079	63	0.0128
63	0.0077	61	0.0128
28	0.0070	28	0.0115
84	0.0069	84	0.0113
85	0.0052	85	0.0086
109	0.0050	109	0.0077
82	0.0043	82	0.0073
91	0.0036	108	0.0060
108	0.0036	91	0.0060
83	0.0032	83	0.0054
94	0.0032	94	0.0053
100	0.0029	100	0.0046
90	0.0029	90	0.0046
12	0.0027	12	0.0044
89	0.0025	89	0.0038
49	0.0023	49	0.0038
102	0.0022	102	0.0036
104	0.0020	104	0.0033
13	0.0019	13	0.0029
67	0.0017	86	0.0027
87	0.0017	87	0.0027
86	0.0016	67	0.0026
68	0.0016	68	0.0026
78	0.0015	78	0.0026
92	0.0015	92	0.0024
110	0.0014	110	0.0023
71	0.0013	16	0.0021
16	0.0013	97	0.0021
27	0.0013	27	0.0021
97	0.0013	71	0.0021
98	0.0012	65	0.0020
65	0.0012	98	0.0020
42	0.0010	42	0.0018
66	0.0010	66	0.0017
20	0.0008	20	0.0013
55	0.0008	55	0.0013
3	0.0008	64	0.0012
64	0.0007	74	0.0012
74	0.0007	3	0.0012
62	0.0007	35	0.0011
35	0.0007	72	0.0011
72	0.0007	37	0.0011
45	0.0006	45	0.0010
96	0.0006	62	0.0009
75	0.0006	75	0.0009
37	0.0005	96	0.0008
1	0.0005	21	0.0007
21	0.0005	1	0.0007
4	0.0004	5	0.0006
5	0.0004	4	0.0006
81	0.0003	81	0.0005
70	0.0003	70	0.0005
95	0.0003	95	0.0004
30	0.0002	15	0.0004
56	0.0002	56	0.0003
15	0.0002	30	0.0003
46	0.0002	46	0.0003
77	0.0002	77	0.0002
73	0.0001	73	0.0002
17	0.0001	17	0.0002
47	0.0001	57	0.0002
93	0.0001	47	0.0001
57	0.0001	93	0.0001
6	0.0000	41	0.0001
18	-0.0000	11	-0.0000
11	-0.0000	19	-0.0000
41	-0.0000	6	-0.0000
19	-0.0000	18	-0.0001
2	-0.0001	2	-0.0001

8	-0.0001	7	-0.0002
7	-0.0001	8	-0.0002
105	-0.0001	105	-0.0002
76	-0.0001	103	-0.0002
25	-0.0002	76	-0.0003
38	-0.0002	38	-0.0003
80	-0.0002	25	-0.0003
103	-0.0002	80	-0.0003
60	-0.0002	60	-0.0003
40	-0.0002	40	-0.0003
23	-0.0002	23	-0.0003
48	-0.0002	48	-0.0004
58	-0.0002	58	-0.0004
88	-0.0002	88	-0.0004
26	-0.0002	34	-0.0004
34	-0.0002	14	-0.0004
51	-0.0003	26	-0.0004
39	-0.0003	53	-0.0005
14	-0.0003	33	-0.0005
53	-0.0003	52	-0.0005
33	-0.0003	39	-0.0005
52	-0.0003	51	-0.0005
69	-0.0003	22	-0.0005
22	-0.0003	24	-0.0005
24	-0.0003	69	-0.0005
54	-0.0003	54	-0.0006
50	-0.0004	50	-0.0007
106	-0.0004	106	-0.0007
29	-0.0004	29	-0.0008
44	-0.0005	44	-0.0008
10	-0.0005	10	-0.0008
59	-0.0005	59	-0.0008
9	-0.0006	9	-0.0009

Mtry=**8** Trees=**12000** Seed=9

Sample-level Error Rate: 0.15
Sample-level Confusion Matrix: (rows = predicted, cols = actual)

18	4
2	16

Variable importance:

Permutation-based Proportion ID	Score	Mean Decrease in Margin (**MDM**) ID	Score
36	0.0238	36	0.0385
99	0.0170	32	0.0279
32	0.0170	99	0.0271
107	0.0131	107	0.0210
79	0.0097	79	0.0161
43	0.0088	43	0.0143
31	0.0088	31	0.0142
101	0.0079	101	0.0129
63	0.0074	63	0.0125
28	0.0073	28	0.0120
61	0.0072	61	0.0120
84	0.0070	84	0.0115
109	0.0053	109	0.0084
85	0.0049	85	0.0080
82	0.0043	82	0.0072
91	0.0039	91	0.0064
90	0.0039	90	0.0061
94	0.0038	94	0.0061
100	0.0033	108	0.0054
108	0.0033	100	0.0053
83	0.0029	83	0.0046
12	0.0027	12	0.0044
102	0.0024	102	0.0039
89	0.0023	49	0.0036
49	0.0022	89	0.0036
86	0.0019	104	0.0032
68	0.0019	86	0.0032
104	0.0018	68	0.0031
78	0.0018	78	0.0030
110	0.0018	110	0.0029

67	0.0017	67	0.0028
87	0.0016	98	0.0027
98	0.0016	66	0.0026
13	0.0015	92	0.0025
66	0.0015	65	0.0024
65	0.0015	13	0.0024
71	0.0014	87	0.0024
92	0.0014	27	0.0023
27	0.0014	42	0.0023
97	0.0014	97	0.0023
42	0.0013	71	0.0023
35	0.0012	35	0.0020
16	0.0011	37	0.0019
37	0.0010	16	0.0018
72	0.0009	72	0.0016
64	0.0009	64	0.0015
74	0.0008	45	0.0014
45	0.0008	74	0.0013
75	0.0007	55	0.0012
55	0.0007	3	0.0011
3	0.0006	75	0.0010
1	0.0006	1	0.0009
62	0.0006	62	0.0008
96	0.0005	20	0.0008
20	0.0005	96	0.0007
21	0.0004	21	0.0007
5	0.0004	2	0.0007
2	0.0004	70	0.0006
70	0.0004	77	0.0006
77	0.0004	81	0.0006
81	0.0003	5	0.0005
93	0.0003	15	0.0004
73	0.0003	73	0.0004
95	0.0003	47	0.0004
47	0.0003	93	0.0004
15	0.0002	95	0.0004
4	0.0002	56	0.0004
57	0.0002	57	0.0004
30	0.0002	4	0.0003
56	0.0002	30	0.0002
46	0.0001	41	0.0002
18	0.0001	46	0.0002
8	0.0001	18	0.0001
41	0.0001	8	0.0001
17	0.0000	17	0.0000
11	0.0000	11	-0.0001
76	-0.0000	76	-0.0001
19	-0.0001	19	-0.0002
6	-0.0001	23	-0.0002
33	-0.0001	22	-0.0002
22	-0.0001	44	-0.0002
48	-0.0001	53	-0.0002
26	-0.0001	33	-0.0002
53	-0.0002	6	-0.0002
103	-0.0002	103	-0.0002
23	-0.0002	26	-0.0002
7	-0.0002	7	-0.0003
51	-0.0002	48	-0.0003
44	-0.0002	105	-0.0003
80	-0.0002	80	-0.0003
58	-0.0002	60	-0.0003
105	-0.0002	58	-0.0004
24	-0.0002	24	-0.0004
60	-0.0002	88	-0.0004
88	-0.0003	51	-0.0004
14	-0.0003	14	-0.0005
52	-0.0003	52	-0.0005
50	-0.0003	40	-0.0005
25	-0.0003	50	-0.0006
40	-0.0004	25	-0.0006
34	-0.0004	34	-0.0006
69	-0.0004	54	-0.0006
38	-0.0004	106	-0.0006
106	-0.0004	69	-0.0007
54	-0.0004	38	-0.0007
39	-0.0005	39	-0.0007
59	-0.0005	10	-0.0008

```
    10   -0.0005          59   -0.0009
     9   -0.0006           9   -0.0009
    29   -0.0006          29   -0.0011
```

Mtry=**6** Trees=12000 Seed=9

```
Sample-level Error Rate: 0.15
Sample-level Confusion Matrix: (rows = predicted, cols = actual)
    18     4
     2    16

Variable importance:
Permutation-based   |   Mean Decrease in
Proportion          |   Margin (MDM)
    ID    Score     |       ID    Score
    36    0.0211          36    0.0341
    99    0.0146          99    0.0235
    32    0.0140          32    0.0225
   107    0.0119         107    0.0192
    79    0.0094          79    0.0151
    31    0.0090          31    0.0146
    43    0.0089          43    0.0142
   101    0.0086         101    0.0141
    61    0.0072          61    0.0117
    28    0.0069          28    0.0116
    63    0.0068          63    0.0113
    84    0.0064          84    0.0107
   109    0.0052          85    0.0083
    85    0.0051         109    0.0081
    82    0.0045          82    0.0073
    94    0.0039          94    0.0065
    90    0.0038         108    0.0062
   108    0.0037          90    0.0061
   100    0.0036         100    0.0059
    83    0.0034          83    0.0057
    91    0.0031          91    0.0053
    89    0.0028          89    0.0044
    49    0.0025         104    0.0043
   104    0.0025          49    0.0042
    12    0.0024         110    0.0039
   110    0.0024          12    0.0039
   102    0.0022         102    0.0037
    68    0.0020          68    0.0031
    87    0.0019          78    0.0030
    78    0.0019          67    0.0029
    86    0.0018          66    0.0029
    66    0.0017          87    0.0029
    27    0.0017          86    0.0028
    67    0.0017          27    0.0026
    71    0.0016          71    0.0026
    13    0.0015          65    0.0026
    65    0.0015          13    0.0024
    97    0.0014          55    0.0024
    92    0.0014          98    0.0024
    55    0.0014          92    0.0023
    98    0.0014          97    0.0023
    42    0.0013          42    0.0023
    16    0.0013          16    0.0021
    75    0.0011          75    0.0019
    35    0.0011          35    0.0019
    72    0.0010          37    0.0018
    64    0.0010          45    0.0018
    45    0.0010          64    0.0017
    37    0.0009          72    0.0016
    74    0.0007          74    0.0012
     3    0.0007           3    0.0011
    21    0.0006          21    0.0011
    93    0.0006          57    0.0009
     1    0.0006           1    0.0009
    77    0.0006          20    0.0009
    20    0.0005          93    0.0009
    95    0.0005          77    0.0009
    57    0.0005          95    0.0007
    70    0.0004          70    0.0007
    30    0.0004          56    0.0007
```

47	0.0004	96	0.0006
56	0.0004	30	0.0006
96	0.0004	47	0.0006
62	0.0004	62	0.0006
81	0.0003	81	0.0005
41	0.0002	41	0.0005
4	0.0002	6	0.0005
6	0.0002	15	0.0004
73	0.0002	4	0.0004
15	0.0002	73	0.0004
5	0.0002	5	0.0002
46	0.0001	7	0.0002
7	0.0001	18	0.0001
18	0.0001	46	0.0001
2	0.0001	8	0.0000
8	0.0001	2	-0.0000
60	-0.0000	60	-0.0001
51	-0.0000	11	-0.0001
19	-0.0001	40	-0.0001
40	-0.0001	19	-0.0001
11	-0.0001	105	-0.0002
105	-0.0001	106	-0.0002
106	-0.0001	17	-0.0002
58	-0.0001	53	-0.0002
25	-0.0001	51	-0.0002
53	-0.0001	25	-0.0002
17	-0.0001	80	-0.0002
80	-0.0002	58	-0.0002
48	-0.0002	48	-0.0002
26	-0.0003	10	-0.0004
76	-0.0003	76	-0.0004
10	-0.0003	88	-0.0004
22	-0.0003	14	-0.0004
52	-0.0003	50	-0.0005
44	-0.0003	22	-0.0005
23	-0.0003	26	-0.0005
88	-0.0003	23	-0.0005
50	-0.0003	38	-0.0005
29	-0.0003	52	-0.0005
14	-0.0003	44	-0.0005
34	-0.0003	33	-0.0005
69	-0.0003	69	-0.0006
33	-0.0003	29	-0.0006
38	-0.0004	103	-0.0006
24	-0.0004	24	-0.0006
103	-0.0004	34	-0.0006
9	-0.0004	9	-0.0007
54	-0.0004	39	-0.0007
39	-0.0004	54	-0.0007
59	-0.0005	59	-0.0009

Mtry=**15** Trees=12000 Seed=9

Sample-level Error Rate: 0.175
Sample-level Confusion Matrix: (rows = predicted, cols = actual)

17	4
3	16

Variable importance:

Permutation-based Proportion | Mean Decrease in Margin (**MDM**)

ID	Score	ID	Score
36	0.0340	36	0.0548
99	0.0215	99	0.0345
32	0.0201	32	0.0325
107	0.0149	107	0.0240
79	0.0115	79	0.0188
43	0.0090	43	0.0146
101	0.0088	101	0.0142
31	0.0085	31	0.0138
63	0.0081	63	0.0136
61	0.0080	61	0.0132
28	0.0064	28	0.0105
84	0.0058	84	0.0096
85	0.0057	85	0.0093
109	0.0048	109	0.0075

82	0.0041	82	0.0070
94	0.0035	94	0.0057
91	0.0034	91	0.0055
108	0.0030	108	0.0050
12	0.0027	12	0.0043
100	0.0027	100	0.0043
90	0.0023	90	0.0038
89	0.0023	83	0.0037
83	0.0022	89	0.0036
49	0.0021	49	0.0035
13	0.0020	13	0.0031
104	0.0018	86	0.0029
86	0.0017	104	0.0029
102	0.0014	102	0.0024
68	0.0014	68	0.0023
78	0.0014	78	0.0022
87	0.0012	67	0.0019
67	0.0012	87	0.0019
20	0.0012	20	0.0019
97	0.0011	110	0.0018
110	0.0011	92	0.0018
92	0.0011	97	0.0018
98	0.0010	98	0.0017
65	0.0010	65	0.0017
66	0.0010	66	0.0016
71	0.0009	16	0.0015
16	0.0009	71	0.0013
27	0.0008	27	0.0013
55	0.0006	42	0.0011
37	0.0006	55	0.0011
74	0.0006	37	0.0011
42	0.0006	74	0.0010
5	0.0006	72	0.0009
96	0.0005	96	0.0008
62	0.0005	5	0.0008
72	0.0004	45	0.0007
3	0.0004	70	0.0007
64	0.0004	64	0.0007
70	0.0004	3	0.0007
45	0.0004	62	0.0007
35	0.0004	56	0.0007
4	0.0004	35	0.0006
1	0.0004	4	0.0006
56	0.0004	1	0.0005
75	0.0002	75	0.0004
21	0.0002	41	0.0003
46	0.0002	81	0.0003
81	0.0002	21	0.0003
95	0.0002	46	0.0002
41	0.0001	95	0.0002
15	0.0001	57	0.0001
93	0.0001	17	0.0001
57	0.0001	15	0.0001
17	0.0001	30	0.0001
30	0.0001	93	0.0001
73	0.0000	73	0.0000
8	0.0000	8	0.0000
88	0.0000	48	0.0000
48	-0.0000	88	-0.0000
19	-0.0000	2	-0.0000
18	-0.0000	19	-0.0001
2	-0.0000	11	-0.0001
11	-0.0001	76	-0.0001
76	-0.0001	18	-0.0001
6	-0.0001	7	-0.0001
7	-0.0001	6	-0.0001
47	-0.0001	22	-0.0001
33	-0.0001	26	-0.0002
23	-0.0001	23	-0.0002
26	-0.0001	60	-0.0002
22	-0.0001	77	-0.0002
60	-0.0001	103	-0.0002
24	-0.0001	33	-0.0002
77	-0.0001	106	-0.0002
58	-0.0002	47	-0.0002
103	-0.0002	105	-0.0002
106	-0.0002	58	-0.0003

54	-0.0002	24	-0.0003
105	-0.0002	54	-0.0003
25	-0.0002	25	-0.0003
14	-0.0002	14	-0.0003
34	-0.0002	34	-0.0003
52	-0.0002	52	-0.0004
10	-0.0002	10	-0.0004
40	-0.0003	50	-0.0004
44	-0.0003	44	-0.0004
80	-0.0003	40	-0.0004
50	-0.0003	51	-0.0005
51	-0.0003	80	-0.0005
38	-0.0003	38	-0.0005
53	-0.0004	39	-0.0007
39	-0.0004	53	-0.0007
59	-0.0004	59	-0.0007
29	-0.0005	29	-0.0008
9	-0.0005	9	-0.0009
69	-0.0006	69	-0.0010

Mtry=**15** Trees=20000 Seed=9

Sample-level Error Rate: 0.175
Sample-level Confusion Matrix: (rows = predicted, cols = actual)

17	4
3	16

Variable importance:

Permutation-based Proportion		Mean Decrease in Margin (**MDM**)	
Permutation-based Proportion		Mean Decrease in Margin (MDM)	
ID	Score	ID	Score
36	0.0344	36	0.0553
99	0.0218	99	0.0350
32	0.0197	32	0.0317
107	0.0153	107	0.0246
79	0.0110	79	0.0182
43	0.0098	43	0.0155
101	0.0085	101	0.0137
61	0.0082	63	0.0135
31	0.0082	61	0.0135
63	0.0080	31	0.0130
28	0.0071	28	0.0118
84	0.0063	84	0.0103
85	0.0056	85	0.0094
109	0.0046	109	0.0071
82	0.0041	82	0.0069
94	0.0034	94	0.0057
91	0.0030	91	0.0051
108	0.0029	108	0.0049
12	0.0029	12	0.0046
100	0.0026	100	0.0042
90	0.0025	90	0.0039
89	0.0025	89	0.0038
83	0.0021	83	0.0035
104	0.0019	104	0.0033
49	0.0019	49	0.0030
13	0.0019	13	0.0029
86	0.0016	86	0.0027
102	0.0015	102	0.0024
68	0.0015	68	0.0024
87	0.0012	87	0.0020
92	0.0012	92	0.0019
67	0.0011	110	0.0019
110	0.0011	66	0.0019
20	0.0011	67	0.0017
66	0.0011	20	0.0017
78	0.0011	78	0.0017
71	0.0011	71	0.0017
97	0.0010	27	0.0016
27	0.0010	16	0.0015
16	0.0009	98	0.0015
65	0.0009	97	0.0015
98	0.0009	65	0.0015

55	0.0007	74	0.0013
74	0.0007	55	0.0013
3	0.0007	64	0.0011
62	0.0007	42	0.0011
64	0.0006	3	0.0011
37	0.0006	37	0.0010
72	0.0006	72	0.0010
42	0.0006	62	0.0008
96	0.0005	35	0.0008
75	0.0005	96	0.0007
35	0.0004	75	0.0007
21	0.0004	21	0.0007
5	0.0004	45	0.0006
1	0.0004	81	0.0006
81	0.0004	56	0.0006
95	0.0003	5	0.0006
45	0.0003	95	0.0006
56	0.0003	1	0.0005
70	0.0002	70	0.0004
93	0.0002	93	0.0003
15	0.0002	41	0.0003
41	0.0001	4	0.0002
4	0.0001	15	0.0002
30	0.0001	30	0.0001
73	0.0000	19	0.0000
46	0.0000	73	-0.0000
19	0.0000	57	-0.0000
57	0.0000	8	-0.0000
8	-0.0000	46	-0.0000
47	-0.0000	18	-0.0001
2	-0.0000	2	-0.0001
18	-0.0000	17	-0.0001
11	-0.0000	23	-0.0001
17	-0.0001	7	-0.0001
7	-0.0001	58	-0.0001
23	-0.0001	11	-0.0001
58	-0.0001	77	-0.0001
77	-0.0001	48	-0.0002
60	-0.0001	60	-0.0002
48	-0.0001	47	-0.0002
6	-0.0001	6	-0.0002
26	-0.0001	26	-0.0002
14	-0.0001	76	-0.0002
51	-0.0001	14	-0.0003
76	-0.0002	51	-0.0003
88	-0.0002	106	-0.0003
106	-0.0002	88	-0.0003
103	-0.0002	103	-0.0003
52	-0.0002	22	-0.0003
25	-0.0002	105	-0.0004
53	-0.0002	52	-0.0004
105	-0.0002	25	-0.0004
22	-0.0002	53	-0.0004
34	-0.0002	34	-0.0004
24	-0.0003	54	-0.0004
80	-0.0003	44	-0.0004
54	-0.0003	24	-0.0004
38	-0.0003	80	-0.0005
44	-0.0003	38	-0.0005
33	-0.0003	50	-0.0006
69	-0.0003	33	-0.0006
40	-0.0004	69	-0.0006
50	-0.0004	39	-0.0006
39	-0.0004	40	-0.0006
29	-0.0004	10	-0.0007
10	-0.0004	29	-0.0008
59	-0.0005	59	-0.0008
9	-0.0005	9	-0.0009

Mtry=**6** Trees=**5000** Seed=9 [die folgende gestrichelte Linie bedeutet nicht, ich bekomme sie nicht weg.em]

Sample-level Error Rate: 0.15
Sample-level Confusion Matrix: (rows = predicted, cols = actual)

18	4
2	16

Variable importance:

Permutation-based Proportion		Mean Decrease in Margin (**MDM**)	
ID	Score	ID	Score
36	0.0224	36	0.0363
32	0.0145	32	0.0233
99	0.0133	99	0.0216
107	0.0122	107	0.0199
79	0.0092	79	0.0149
31	0.0089	31	0.0144
43	0.0089	43	0.0139
101	0.0078	101	0.0129
61	0.0077	61	0.0126
28	0.0071	28	0.0118
63	0.0068	63	0.0111
84	0.0064	84	0.0105
109	0.0052	109	0.0082
82	0.0045	91	0.0076
91	0.0045	82	0.0073
94	0.0043	94	0.0071
85	0.0043	85	0.0069
90	0.0039	90	0.0063
108	0.0036	108	0.0062
100	0.0035	104	0.0057
104	0.0033	100	0.0054
12	0.0029	83	0.0049
110	0.0029	12	0.0048
83	0.0029	110	0.0046
49	0.0026	49	0.0044
89	0.0024	89	0.0043
102	0.0021	102	0.0035
78	0.0021	65	0.0034
87	0.0020	71	0.0033
71	0.0020	78	0.0033
67	0.0020	67	0.0033
65	0.0020	68	0.0031
68	0.0019	87	0.0030
27	0.0019	27	0.0029
97	0.0017	97	0.0029
92	0.0015	98	0.0023
66	0.0015	92	0.0023
98	0.0013	66	0.0022
3	0.0013	77	0.0020
16	0.0012	42	0.0019
86	0.0012	45	0.0019
13	0.0012	86	0.0019
42	0.0011	13	0.0019
77	0.0011	16	0.0018
72	0.0011	37	0.0018
45	0.0010	72	0.0017
74	0.0010	55	0.0017
1	0.0010	3	0.0017
64	0.0009	74	0.0016
37	0.0009	35	0.0015
55	0.0009	64	0.0015
35	0.0009	1	0.0014
51	0.0007	20	0.0011
20	0.0007	51	0.0011
93	0.0006	4	0.0008
21	0.0005	57	0.0008
4	0.0005	21	0.0008
96	0.0005	6	0.0007
75	0.0005	96	0.0007
30	0.0005	93	0.0007
6	0.0004	75	0.0007
57	0.0004	30	0.0007
56	0.0004	105	0.0006
62	0.0004	56	0.0006
8	0.0004	95	0.0005
47	0.0003	62	0.0005
5	0.0003	8	0.0005
70	0.0003	47	0.0005
95	0.0003	5	0.0004
105	0.0002	41	0.0004
41	0.0002	70	0.0004
40	0.0002	40	0.0003
81	0.0002	81	0.0003
7	0.0002	48	0.0003

48	0.0001	18	0.0003
18	0.0001	7	0.0002
15	0.0001	11	0.0001
11	0.0000	15	0.0001
60	0.0000	60	0.0000
25	0.0000	106	0.0000
46	0.0000	80	-0.0000
73	-0.0000	25	-0.0000
58	-0.0000	46	-0.0000
80	-0.0000	73	-0.0000
19	-0.0000	19	-0.0001
106	-0.0001	58	-0.0001
52	-0.0001	52	-0.0002
33	-0.0001	33	-0.0002
76	-0.0001	14	-0.0002
44	-0.0002	76	-0.0002
69	-0.0002	38	-0.0003
53	-0.0002	23	-0.0003
38	-0.0002	88	-0.0003
23	-0.0002	69	-0.0003
88	-0.0002	44	-0.0004
14	-0.0002	53	-0.0004
2	-0.0002	17	-0.0004
17	-0.0003	22	-0.0006
22	-0.0003	2	-0.0006
50	-0.0003	50	-0.0006
103	-0.0004	34	-0.0007
24	-0.0004	103	-0.0007
34	-0.0004	24	-0.0007
9	-0.0004	9	-0.0008
59	-0.0005	26	-0.0008
29	-0.0005	59	-0.0008
26	-0.0005	39	-0.0009
39	-0.0006	29	-0.0009
54	-0.0006	54	-0.0010
10	-0.0007	10	-0.0012

Mtry=6 Trees=2**000** Seed=9

Sample-level Error Rate: 0.15

Sample-level Confusion Matrix: (rows = predicted, cols = actual)

18	4
2	16

Variable importance:

Permutation-based Proportion		Mean Decrease in Margin (**MDM**)	
ID	Score	ID	Score
36	0.0236	36	0.0380
107	0.0129	107	0.0216
99	0.0123	99	0.0198
32	0.0118	32	0.0191
31	0.0101	31	0.0167
79	0.0093	79	0.0154
43	0.0089	43	0.0145
101	0.0084	101	0.0141
63	0.0082	63	0.0138
28	0.0082	28	0.0135
61	0.0074	61	0.0120
84	0.0064	84	0.0104
109	0.0054	82	0.0089
82	0.0050	109	0.0088
90	0.0049	85	0.0077
85	0.0047	90	0.0077
83	0.0046	83	0.0075
108	0.0039	108	0.0066
100	0.0036	91	0.0058
94	0.0035	100	0.0056
91	0.0032	94	0.0056
104	0.0028	78	0.0048
78	0.0028	104	0.0047
86	0.0026	110	0.0041
12	0.0025	12	0.0041
110	0.0024	102	0.0041
65	0.0023	86	0.0040

49	0.0022	65	0.0036
89	0.0022	49	0.0036
102	0.0021	89	0.0035
87	0.0021	66	0.0031
66	0.0020	87	0.0030
98	0.0019	27	0.0030
27	0.0018	98	0.0030
68	0.0017	37	0.0029
92	0.0017	92	0.0029
45	0.0017	68	0.0028
3	0.0016	67	0.0028
71	0.0016	45	0.0027
37	0.0016	97	0.0027
67	0.0016	71	0.0025
97	0.0015	13	0.0025
13	0.0014	3	0.0025
42	0.0012	16	0.0021
16	0.0012	42	0.0020
72	0.0011	72	0.0019
1	0.0011	55	0.0018
55	0.0010	1	0.0017
74	0.0009	35	0.0015
35	0.0008	77	0.0014
77	0.0008	74	0.0014
75	0.0007	20	0.0013
93	0.0007	75	0.0012
20	0.0007	56	0.0011
56	0.0006	21	0.0011
21	0.0006	64	0.0011
64	0.0006	5	0.0010
5	0.0006	93	0.0009
96	0.0005	47	0.0009
47	0.0005	81	0.0007
4	0.0005	4	0.0007
81	0.0004	15	0.0007
73	0.0004	96	0.0006
15	0.0003	51	0.0005
62	0.0003	6	0.0005
51	0.0003	105	0.0005
105	0.0002	62	0.0004
8	0.0002	7	0.0004
30	0.0002	41	0.0003
41	0.0002	25	0.0003
6	0.0002	106	0.0003
70	0.0002	8	0.0003
25	0.0002	57	0.0003
106	0.0002	73	0.0003
19	0.0002	19	0.0003
7	0.0001	30	0.0002
57	0.0001	18	0.0002
46	0.0001	70	0.0002
18	0.0001	60	0.0002
60	0.0000	69	0.0001
69	0.0000	14	0.0000
33	0.0000	76	0.0000
38	-0.0000	46	-0.0000
40	-0.0000	40	-0.0000
14	-0.0000	11	-0.0001
58	-0.0001	58	-0.0001
76	-0.0001	48	-0.0001
23	-0.0001	23	-0.0002
11	-0.0001	38	-0.0002
52	-0.0002	95	-0.0002
48	-0.0002	29	-0.0002
29	-0.0002	52	-0.0003
95	-0.0002	33	-0.0003
53	-0.0002	17	-0.0004
103	-0.0002	53	-0.0004
17	-0.0002	88	-0.0005
80	-0.0003	103	-0.0005
50	-0.0003	80	-0.0006
44	-0.0003	44	-0.0006
34	-0.0004	50	-0.0007
88	-0.0004	59	-0.0007
2	-0.0004	34	-0.0008
59	-0.0004	26	-0.0009
9	-0.0005	39	-0.0009

```
    10   -0.0006           2   -0.0009
    26   -0.0006          22   -0.0010
    39   -0.0006          10   -0.0010
    22   -0.0006           9   -0.0011
    24   -0.0007          24   -0.0011
    54   -0.0008          54   -0.0013
```

Mtry=6 Trees=**1000** Seed=9

```
Sample-level Error Rate: 0.15
Sample-level Confusion Matrix: (rows = predicted, cols = actual)
    18     4
     2    16
```

Variable importance:

```
Permutation-based   |   Mean Decrease in
Proportion          |   Margin (MDM)
    ID    Score     |       ID    Score
    36    0.0236          36    0.0376
   107    0.0144         107    0.0241
    32    0.0122          32    0.0193
    79    0.0119          79    0.0185
    31    0.0108          31    0.0179
    99    0.0105          99    0.0174
    43    0.0090          28    0.0143
    84    0.0086          84    0.0142
    61    0.0084          43    0.0139
    28    0.0081          61    0.0137
   101    0.0067         101    0.0109
    63    0.0062          63    0.0098
    85    0.0055          85    0.0084
   108    0.0051          91    0.0082
    82    0.0050          82    0.0082
   109    0.0046         108    0.0079
    91    0.0046         109    0.0077
    83    0.0044          83    0.0070
   104    0.0038         104    0.0060
    90    0.0036          90    0.0060
    89    0.0029          89    0.0051
    87    0.0028          71    0.0050
    71    0.0028          87    0.0048
    12    0.0027         102    0.0046
    65    0.0027         110    0.0046
    78    0.0027          86    0.0045
   110    0.0026          65    0.0044
    86    0.0026          12    0.0044
   102    0.0025          78    0.0041
   100    0.0023          94    0.0035
    94    0.0023         100    0.0035
    66    0.0022          49    0.0034
    98    0.0021          72    0.0034
    72    0.0021          66    0.0032
    49    0.0020          98    0.0032
     1    0.0017          92    0.0028
    92    0.0017           1    0.0028
    67    0.0017          68    0.0028
     3    0.0016           3    0.0027
    55    0.0015          27    0.0026
    68    0.0015          55    0.0026
    27    0.0015          67    0.0025
     4    0.0014          45    0.0023
    45    0.0013          13    0.0022
    13    0.0013          20    0.0022
    75    0.0013           4    0.0021
    70    0.0013          70    0.0021
    20    0.0012          75    0.0020
    64    0.0012          69    0.0020
    69    0.0011          77    0.0019
    35    0.0009          64    0.0018
    74    0.0009          35    0.0016
    77    0.0009          74    0.0014
    42    0.0008          19    0.0014
    93    0.0008          42    0.0014
    97    0.0008          97    0.0013
    19    0.0007          37    0.0011
    16    0.0006          93    0.0011
```

```
    96    0.0006          56    0.0008
    37    0.0005          16    0.0008
    56    0.0005          96    0.0007
    51    0.0004          51    0.0007
    33    0.0003          57    0.0006
     5    0.0003           5    0.0006
    53    0.0003          53    0.0005
    21    0.0003          41    0.0005
    57    0.0003          33    0.0004
   105    0.0003          21    0.0004
    41    0.0002         105    0.0004
    76    0.0002          81    0.0003
     8    0.0001          76    0.0003
    73    0.0001           8    0.0002
    81    0.0001          47    0.0002
    26    0.0001          11    0.0001
    47    0.0001          15    0.0001
    14    0.0001          52    0.0001
    34    0.0000          14    0.0001
    52    0.0000          62    0.0000
    30    0.0000          88    0.0000
    46    0.0000          25   -0.0000
   103    0.0000          26   -0.0000
    62    0.0000         106   -0.0000
   106   -0.0000          73   -0.0001
    25   -0.0000          34   -0.0001
     7   -0.0001          30   -0.0001
    58   -0.0001          58   -0.0002
    11   -0.0001           6   -0.0002
    15   -0.0001         103   -0.0002
    88   -0.0002           7   -0.0003
    24   -0.0002          46   -0.0003
    18   -0.0002          60   -0.0004
    60   -0.0002          18   -0.0004
    17   -0.0003          24   -0.0004
     6   -0.0003          17   -0.0005
    38   -0.0003          48   -0.0005
    40   -0.0003          38   -0.0006
    48   -0.0003          80   -0.0007
     2   -0.0004          40   -0.0007
    80   -0.0005          22   -0.0008
    44   -0.0005          54   -0.0009
    95   -0.0005          95   -0.0010
    54   -0.0006          59   -0.0010
    50   -0.0006          50   -0.0010
    59   -0.0006           2   -0.0010
    22   -0.0006          29   -0.0011
    23   -0.0007          44   -0.0011
    29   -0.0007          23   -0.0012
    39   -0.0007          39   -0.0012
     9   -0.0008           9   -0.0014
    10   -0.0016          10   -0.0028
```

Mtry=6 Trees=**12000** Seed=9

```
Sample-level Error Rate: 0.15
Sample-level Confusion Matrix: (rows = predicted, cols = actual)
    18     4
     2    16

Variable importance:
Permutation-based   |   Mean Decrease in
Proportion          |   Margin (MDM)
    ID    Score     |       ID    Score
    36    0.0211          36    0.0341
    99    0.0146          99    0.0235
    32    0.0140          32    0.0225
   107    0.0119         107    0.0192
    79    0.0094          79    0.0151
    31    0.0090          31    0.0146
    43    0.0089          43    0.0142
   101    0.0086         101    0.0141
    61    0.0072          61    0.0117
    28    0.0069          28    0.0116
    63    0.0068          63    0.0113
```

84	0.0064	84	0.0107
109	0.0052	85	0.0083
85	0.0051	109	0.0081
82	0.0045	82	0.0073
94	0.0039	94	0.0065
90	0.0038	108	0.0062
108	0.0037	90	0.0061
100	0.0036	100	0.0059
83	0.0034	83	0.0057
91	0.0031	91	0.0053
89	0.0028	89	0.0044
49	0.0025	104	0.0043
104	0.0025	49	0.0042
12	0.0024	110	0.0039
110	0.0024	12	0.0039
102	0.0022	102	0.0037
68	0.0020	68	0.0031
87	0.0019	78	0.0030
78	0.0019	67	0.0029
86	0.0018	66	0.0029
66	0.0017	87	0.0029
27	0.0017	86	0.0028
67	0.0017	27	0.0026
71	0.0016	71	0.0026
13	0.0015	65	0.0026
65	0.0015	13	0.0024
97	0.0014	55	0.0024
92	0.0014	98	0.0024
55	0.0014	92	0.0023
98	0.0014	97	0.0023
42	0.0013	42	0.0023
16	0.0013	16	0.0021
75	0.0011	75	0.0019
35	0.0011	35	0.0019
72	0.0010	37	0.0018
64	0.0010	45	0.0018
45	0.0010	64	0.0017
37	0.0009	72	0.0016
74	0.0007	74	0.0012
3	0.0007	3	0.0011
21	0.0006	21	0.0011
93	0.0006	57	0.0009
1	0.0006	1	0.0009
77	0.0006	20	0.0009
20	0.0005	93	0.0009
95	0.0005	77	0.0009
57	0.0005	95	0.0007
70	0.0004	70	0.0007
30	0.0004	56	0.0007
47	0.0004	96	0.0006
56	0.0004	30	0.0006
96	0.0004	47	0.0006
62	0.0004	62	0.0006
81	0.0003	81	0.0005
41	0.0002	41	0.0005
4	0.0002	6	0.0005
6	0.0002	15	0.0004
73	0.0002	4	0.0004
15	0.0002	73	0.0004
5	0.0002	5	0.0002
46	0.0001	7	0.0002
7	0.0001	18	0.0001
18	0.0001	46	0.0001
2	0.0001	8	0.0000
8	0.0001	2	-0.0000
60	-0.0000	60	-0.0001
51	-0.0000	11	-0.0001
19	-0.0001	40	-0.0001
40	-0.0001	19	-0.0001
11	-0.0001	105	-0.0002
105	-0.0001	106	-0.0002
106	-0.0001	17	-0.0002
58	-0.0001	53	-0.0002
25	-0.0001	51	-0.0002
53	-0.0001	25	-0.0002
17	-0.0001	80	-0.0002
80	-0.0002	58	-0.0002

48	-0.0002	48	-0.0002
26	-0.0003	10	-0.0004
76	-0.0003	76	-0.0004
10	-0.0003	88	-0.0004
22	-0.0003	14	-0.0004
52	-0.0003	50	-0.0005
44	-0.0003	22	-0.0005
23	-0.0003	26	-0.0005
88	-0.0003	23	-0.0005
50	-0.0003	38	-0.0005
29	-0.0003	52	-0.0005
14	-0.0003	44	-0.0005
34	-0.0003	33	-0.0005
69	-0.0003	69	-0.0006
33	-0.0003	29	-0.0006
38	-0.0004	103	-0.0006
24	-0.0004	24	-0.0006
103	-0.0004	34	-0.0006
9	-0.0004	9	-0.0007
54	-0.0004	39	-0.0007
39	-0.0004	54	-0.0007
59	-0.0005	59	-0.0009

Mtry=6 Trees=**20000** Seed=9

Sample-level Error Rate: 0.15
Sample-level Confusion Matrix: (rows = predicted, cols = actual)

18	4
2	16

Variable importance:

Permutation-based Proportion ID	Score	Mean Decrease in Margin (**MDM**) ID	Score
36	0.0213	36	0.0344
99	0.0144	99	0.0235
32	0.0143	32	0.0232
107	0.0120	107	0.0194
79	0.0093	79	0.0151
43	0.0087	101	0.0141
101	0.0086	43	0.0138
31	0.0080	31	0.0130
28	0.0074	28	0.0123
61	0.0070	63	0.0118
63	0.0070	61	0.0115
84	0.0065	84	0.0107
109	0.0056	109	0.0088
85	0.0053	85	0.0086
82	0.0044	82	0.0073
90	0.0037	108	0.0062
108	0.0037	94	0.0059
94	0.0037	91	0.0059
91	0.0035	90	0.0059
100	0.0034	83	0.0057
83	0.0033	100	0.0054
12	0.0027	12	0.0044
49	0.0027	49	0.0043
89	0.0026	102	0.0042
102	0.0025	104	0.0042
104	0.0025	89	0.0041
27	0.0021	110	0.0035
87	0.0021	27	0.0033
68	0.0021	68	0.0033
110	0.0021	87	0.0032
86	0.0019	66	0.0032
67	0.0019	65	0.0031
78	0.0018	86	0.0031
66	0.0018	67	0.0031
65	0.0018	78	0.0030
71	0.0018	71	0.0030
97	0.0017	97	0.0029
98	0.0016	98	0.0028
42	0.0016	42	0.0026
13	0.0015	13	0.0024
16	0.0013	55	0.0022

92	0.0013	16	0.0022
55	0.0013	92	0.0021
75	0.0012	75	0.0020
35	0.0011	35	0.0019
37	0.0009	37	0.0017
72	0.0009	72	0.0015
74	0.0008	45	0.0015
45	0.0008	20	0.0013
20	0.0008	74	0.0013
64	0.0007	64	0.0012
77	0.0007	77	0.0012
1	0.0007	1	0.0011
3	0.0007	3	0.0010
93	0.0006	93	0.0008
96	0.0005	96	0.0008
62	0.0005	70	0.0008
70	0.0005	81	0.0008
21	0.0005	21	0.0007
81	0.0005	73	0.0007
56	0.0004	62	0.0007
73	0.0004	56	0.0007
57	0.0003	57	0.0006
15	0.0003	41	0.0006
95	0.0003	15	0.0006
41	0.0003	95	0.0005
5	0.0003	18	0.0005
30	0.0003	5	0.0004
18	0.0002	30	0.0004
6	0.0002	6	0.0004
4	0.0002	4	0.0003
47	0.0001	47	0.0002
2	0.0001	7	0.0001
46	0.0001	2	0.0001
7	0.0000	19	-0.0000
19	0.0000	60	-0.0000
17	-0.0000	17	-0.0000
60	-0.0000	46	-0.0000
8	-0.0000	40	-0.0001
40	-0.0000	8	-0.0001
51	-0.0000	11	-0.0001
58	-0.0001	106	-0.0001
11	-0.0001	105	-0.0002
105	-0.0001	33	-0.0002
33	-0.0001	58	-0.0002
48	-0.0001	51	-0.0002
106	-0.0001	48	-0.0002
53	-0.0002	53	-0.0002
44	-0.0002	80	-0.0003
76	-0.0002	76	-0.0003
80	-0.0002	44	-0.0003
26	-0.0002	26	-0.0004
38	-0.0002	38	-0.0004
25	-0.0003	14	-0.0004
24	-0.0003	25	-0.0004
14	-0.0003	24	-0.0004
54	-0.0003	88	-0.0005
23	-0.0003	54	-0.0005
88	-0.0003	23	-0.0005
52	-0.0003	52	-0.0005
34	-0.0003	22	-0.0006
22	-0.0004	50	-0.0006
50	-0.0004	34	-0.0006
69	-0.0004	10	-0.0006
103	-0.0004	103	-0.0007
10	-0.0004	29	-0.0008
29	-0.0004	69	-0.0008
9	-0.0005	59	-0.0008
39	-0.0005	39	-0.0009

Mtry=6 Trees=**40000** Seed=9

```
Sample-level Error Rate: 0.15
Sample-level Confusion Matrix: (rows = predicted, cols = actual)
    18     4
     2    16
```

Variable importance:

Permutation-based Proportion ID	Score	Mean Decrease in Margin (**MDM**) ID	Score
36	0.0221	36	0.0355
99	0.0148	99	0.0239
32	0.0147	32	0.0238
107	0.0119	107	0.0191
79	0.0092	79	0.0150
43	0.0089	43	0.0141
101	0.0084	101	0.0139
31	0.0081	31	0.0132
61	0.0071	61	0.0116
28	0.0070	63	0.0116
63	0.0069	28	0.0115
84	0.0066	84	0.0108
109	0.0054	109	0.0083
85	0.0050	85	0.0081
82	0.0048	82	0.0080
108	0.0039	108	0.0066
90	0.0038	90	0.0061
100	0.0037	100	0.0060
91	0.0034	91	0.0056
83	0.0033	83	0.0056
94	0.0033	94	0.0053
89	0.0029	89	0.0046
104	0.0027	104	0.0046
49	0.0027	49	0.0044
12	0.0025	102	0.0039
110	0.0023	12	0.0039
102	0.0023	110	0.0038
86	0.0022	86	0.0035
87	0.0021	78	0.0034
78	0.0020	87	0.0032
68	0.0019	66	0.0032
71	0.0019	68	0.0031
65	0.0019	65	0.0031
66	0.0019	71	0.0031
67	0.0017	97	0.0027
27	0.0017	27	0.0027
97	0.0017	67	0.0027
98	0.0015	98	0.0025
13	0.0014	42	0.0023
92	0.0014	92	0.0023
42	0.0013	55	0.0022
55	0.0013	16	0.0022
16	0.0013	13	0.0021
35	0.0011	35	0.0020
37	0.0010	37	0.0019
75	0.0010	75	0.0017
72	0.0010	72	0.0017
74	0.0010	64	0.0015
64	0.0009	45	0.0015
45	0.0009	74	0.0015
3	0.0008	20	0.0013
20	0.0008	3	0.0013
1	0.0007	1	0.0010
70	0.0006	70	0.0010
77	0.0005	77	0.0009
62	0.0005	56	0.0008
96	0.0005	81	0.0007
56	0.0005	96	0.0007
93	0.0004	62	0.0007
81	0.0004	93	0.0006
21	0.0003	57	0.0005
5	0.0003	21	0.0005
47	0.0003	41	0.0005
30	0.0003	73	0.0005
15	0.0003	15	0.0004
57	0.0003	5	0.0004
73	0.0003	30	0.0004
4	0.0003	47	0.0004
95	0.0003	95	0.0004
41	0.0002	4	0.0003
46	0.0002	6	0.0003
18	0.0002	18	0.0002
6	0.0002	46	0.0002

8	0.0001	8	0.0001
11	0.0001	11	0.0000
51	0.0000	51	0.0000
17	0.0000	17	0.0000
19	-0.0000	7	-0.0000
7	-0.0000	19	-0.0001
60	-0.0000	60	-0.0001
2	-0.0000	105	-0.0001
105	-0.0001	2	-0.0001
53	-0.0001	53	-0.0002
48	-0.0001	48	-0.0002
58	-0.0001	58	-0.0002
52	-0.0002	80	-0.0003
38	-0.0002	52	-0.0003
80	-0.0002	38	-0.0003
88	-0.0002	14	-0.0003
26	-0.0002	103	-0.0004
14	-0.0002	88	-0.0004
103	-0.0002	26	-0.0004
25	-0.0002	25	-0.0004
76	-0.0003	76	-0.0004
50	-0.0003	50	-0.0004
33	-0.0003	33	-0.0004
34	-0.0003	40	-0.0005
24	-0.0003	24	-0.0005
40	-0.0003	106	-0.0005
22	-0.0003	23	-0.0005
10	-0.0003	10	-0.0005
106	-0.0003	22	-0.0005
23	-0.0003	54	-0.0005
54	-0.0003	34	-0.0005
69	-0.0004	44	-0.0006
44	-0.0004	69	-0.0007
29	-0.0004	39	-0.0007
39	-0.0004	9	-0.0008
9	-0.0005	59	-0.0008
59	-0.0005	29	-0.0008

ENDE****************
